# Facial nerve involvement in critical illness polyneuropathy

**DOI:** 10.4103/0019-5049.71038

**Published:** 2010

**Authors:** Mohan Gurjar, Afzal Azim, Arvind K Baronia, Banani Poddar

**Affiliations:** Department of Critical Care Medicine, Sanjay Gandhi Post Graduate Institute of Medical Sciences, Lucknow, India

**Keywords:** Critical illness, facial nerve, polyneuropathy

## Abstract

Although ICU-acquired neuromuscular weakness is a well-known problem, critical illness neuropathy is an under-diagnosed entity in critically ill patients. Facial musculature is typically not involved in critical illness neuropathy. This report highlights an unusual presentation of critical illness polyneuropathy in a patient with involvement of facial musculature.

## INTRODUCTION

Although described in literature since the early 1980s, critical illness neuropathy is a common, but under-diagnosed entity in critically ill patients. It occurs as a complication in 50 – 70% of the patients with severe sepsis.[1] Sepsis, multi-organ failure, and length of stay in the Intensive Care Unit (ICU) have been traditionally thought to predispose to critical illness polyneuropathy (CIP). Diagnostic criteria for critical illness polyneuropathy, as described by Bolton, include, (i) the patient is critically ill, (ii) difficulty in weaning from ventilator after exclusion of non-neuromuscular causes such as heart and lung disease, (iii) possible limb weakness, and (iv) electrophysiological evidence of axonal motor and sensory polyneuropathy.[1] We report an unusual presentation of critical illness neuropathy with facial involvement.

## CASE REPORT

A 45-year-old male presented to the Emergency Room with complaints of fever, with cough and expectoration, for five days and difficulty in breathing for two days. He was a known hypertensive, controlled with tablet amlodipine 5 mg, once a day, for four years, and he also had chronic pancreatitis for the last two years. On clinical examination he was of average build, conscious, oriented, and moving all limbs. He had tachypnoea (> 35 / minute) with respiratory distress and tachycardia (heart rate 118 / minute). Chest auscultation revealed bilateral fine crepitations with occasional ronchi. Arterial blood gas was suggestive of acute respiratory distress syndrome (ARDS) (PaO_2_/ FiO_2_ ratios < 200 and PaCO_2_-23.mmHg). Orotracheal intubation was carried out and the patient was kept on mechanical ventilation (Maquet ventilator, mode SIMV, with pressure control), and then shifted to the ICU. Early laboratory reports revealed hypokalemia (Serum K^+^ 2.8 meq/L), hyperglycemia (250 mg/dl), and leucocytosis (TLC-14000). A chest X-ray showed bilateral basal consolidation. Other investigations were within normal limits. His provisional diagnosis was pneumonia with ARDS, with dyselectrolytemia. After collecting the appropriate samples (endotracheal aspirate, blood) for cultures, antibiotics were started, along with other supportive care. Hypokalemia and hyperglycemia were corrected with intravenous KCl and insulin infusion, respectively. He was adequately sedated with midazolam infusion and remained haemodynamically stable. By the end of the first week there was complete resolution of infiltrates on the chest X-ray, hence, a spontaneous breathing trial was given, which exhibited poor spontaneous respiratory efforts and weak cough reflex. He had ill sustained handgrip and neck holding was absent. It was anticipated that he might require prolonged mechanical ventilation; therefore, percutaneous dilatational tracheostomy was done. A detailed neurological examination revealed quadriparesis with depressed deep tendon reflexes and bilateral facial weakness, while the other cranial nerves were normal. He did not receive any muscle relaxant or steroid during his stay. His serum electrolytes (Na, K, Ca, Mg and phosphates) were within normal limits. A cerebrospinal fluid (CSF) examination was conducted on the eleventh day of admission, which showed a normal picture (clear, 6 cells/µL, protein 41 mg/dL, glucose 56 mg/dL). Magnetic resonance imaging (MRI) of the brain was also normal. Urine analysis for porphobilinogen was negative. An electromyography and nerve conduction study revealed reduced motor and sensory action potential, with normal conduction velocities, suggestive of critical illness polyneuropathy (CIP). As no specific management exists for CIP, and patients recover spontaneously over a period of time, we managed him by controlling of the sepsis, good glycemic levels and electrolyte homeostasis, and physiotherapy and nutrition. He was weaned off from the ventilator in the next two weeks. His limb weakness and facial weakness improved over the next three weeks and he recovered to the extent of performing daily routine activities independently after three months.

## DISCUSSION

The usual clinical picture of CIP consists of difficulty in weaning from mechanical ventilation, quadriparesis, which is often accompanied by muscle wasting of the limbs.[1] The tendon reflexes are mostly absent or depressed. Facial musculature is often strikingly spared.[2] Usually the onset and recovery of critical illness polyneuropathy takes a few weeks, but in literature some case reports of early onset and recovery are also reported.[3,4] Laboratory investigations are nonspecific. The neurophysiological examination shows an axonal polyneuropathy and sometimes myopathic altered motor unit potentials.

Differential diagnosis of acute neuromuscular weakness commonly includes the Guillain-Barre syndrome (GBS), critical illness neuropathy and myopathy, metabolic neuropathies, toxic neuropathies, and neuropathies due to nutritional deficiencies. In our patient neuropathies due to metabolic origin were ruled out (hypophosphatemia and porphyria). Neuropathy due to nutritional deficiency was unlikely because of a short course of illness and he did not have any clinical or laboratory evidence of chronic malnutrition.

Differentiating CIP from GBS may be quite difficult on purely clinical grounds, as GBS is known for diverse and atypical presentations. The nerve conduction velocity test shows decreased velocity in typical GBS; while a feature of axonal neuropathy, that is, the normal conduction velocity and decreased action potential seen in CIP as well as in the axonal form of GBS (10 – 20% of all GBS). The major clinical difference between CIP and axonal GBS is that CIP is a part of the critical illness that occurs during the ICU stay, whereas, axonal GBS is a severe form of GBS, in a patient with typical antecedents of GBS.[5] A CSF finding at one week into the course of illness can also differentiate between these two diseases. Furthermore, a nerve biopsy and detection of an anti-ganglioside antibody could be helpful in differentiating CIP from axonal GBS.[5] It is important to differentiate, as immunoglobulin is required in GBS, while supportive care is needed in CIP, for a better outcome.

However, in our patient the involvement of facial weakness was suggestive of GB syndrome, as facial weakness is not a reported feature of critical illness neuropathy–myopathy. However, the clinical presentation, along with a CSF examination, supported by neurophysiological investigations, was more in favour of critical illness polyneuropathy. The possibility of myopathy due to steroid was ruled out as the patient did not receive any steroid; although co-existing critical illness myopathy (CIM) could not be ruled out completely due to lack of availability of specialised tests, such as, direct muscle stimulation (DMS), quantitative electromyography (QEMG), and motor unit number estimation (MUNE), in our hospital.[6]

The exact pathogenesis for the neurological effects in CIP is not known. In a recent review Latronico *et al*. has described the pathophysiology of CIP and CIM as a complex, involving metabolic, inflammatory and bioenergetic alterations.[7] The severity of the polyneuropathy depends on the length of stay in the ICU, as also with increasing serum glucose and decreasing serum albumin concentrations.[1] Endoneural oedema promoted by hyperglycemia and hypoalbuminemia can induce hypoxia by an increase in intercapillary distance and other mechanisms.[1] Cytokines and inflammatory mediators also play an important role. In recent times, a low molecular weight toxin has been identified in the serum of CIP patients.[8] It will be quite premature to state that similar mechanisms may also involve the cranial nerves. A greater review of the pathogenesis and evidence will be required to document that cranial nerves may also be involved in critical illness polyneuropathy, as seen in our patient.

Although uncommon, facial musculature may be involved in critical illness polyneuropathy and clinicians should also make a careful differential to rule out other causes. A complete neurological examination from time to time, supplemented with history, clinical presentation, and electrophysiological studies, is the key to timely diagnosis, interventions, and prognostification.
